# Supplementary material for: The advantages of abdominal compression with shallow breathing during left-sided postmastectomy radiotherapy by Helical TomoTherapy
Source: PLoS One. 2021 Jul 16;16(7):e0254934. doi: 10.1371/journal.pone.0254934 (PMC8284651; doi:10.1371/journal.pone.0254934)
Supplement: S1 Table — (DOCX) [file pone.0254934.s001.docx]

S1 Table Dose Constraints to the PTV and OARs

| Targets | Dose constraints |
| --- | --- |
| PTV50 | D50% ≥ 100%  V47.5 ≥ 95%  V57.5 < 2% |
| Ipsilateral lung | V20_Gy_ < 30%  V30_Gy_ < 20% |
| Contralateral lung | V5_Gy_ < 20% |
| Contralateral breast | V5_Gy_ < 10%  D_2%_ < 8 Gy |
| Heart | D_mean_ < 10 Gy  V20_Gy_ < 15% |
| LAD | D_2%_ <50 Gy |
| Spinal cord | D_2%_ <20 Gy |
