# Supplementary material for: The advantages of abdominal compression with shallow breathing during left-sided postmastectomy radiotherapy by Helical TomoTherapy
Source: PLoS One. 2021 Jul 16;16(7):e0254934. doi: 10.1371/journal.pone.0254934 (PMC8284651; doi:10.1371/journal.pone.0254934)
Supplement: Attachment — Submitted filename: Review Comments.docx [file pone.0254934.s003.docx]

I am happy to see the authors have addressed my questions. Some minor problems:

1. Line 153-155, it is not clear to me how the sample size of 21 is determined with using various \rho values. Please specify.
2. Line 183-184, ' Moreover, using ACSB resulted in a significant decrease in mean heart dose (MHD) with 9.17 vs 1839.81 Gy (p<0.0001) and maximum heart dose with 43.79 vs 45.45 Gy (p=0.0144).' I think here more accurately, the 'mean heart dose' should be the mean of the mean heart dose and the 'maximum heart dose' is 'the mean of the maximum heart dose'.
3. Line 187, 'no difference in the LAD volume' should be 'no statistical difference in the mean LAD volume'. There are some other places with similar problems that I would suggest the authors consider for a more accurate expression.
4. Figure 2, please add units to x and y axis labels.
