# Supplementary material for: The advantages of abdominal compression with shallow breathing during left-sided postmastectomy radiotherapy by Helical TomoTherapy
Source: PLoS One. 2021 Jul 16;16(7):e0254934. doi: 10.1371/journal.pone.0254934 (PMC8284651; doi:10.1371/journal.pone.0254934)
Supplement: Attachment — Submitted filename: Response to reviewer-2 (minor revision).docx [file pone.0254934.s004.docx]

June 21, 2021

Dear Dr. Jennifer Wei Zou and reviewers,

Thank you very much for you kindly suggestion. We carefully considered all the comments. I have highlighted the changes within the manuscript based on those comments by using “Track Changes” option in Microsoft Word. Here is a point-by-point response to the reviewer’s comments and recommendations.

**Journal Requirements:**

Please review your reference list to ensure that it is complete and correct. If you have cited papers that have been retracted, please include the rationale for doing so in the manuscript text, or remove these references and replace them with relevant current references. Any changes to the reference list should be mentioned in the rebuttal letter that accompanies your revised manuscript. If you need to cite a retracted article, indicate the article’s retracted status in the References list and also include a citation and full reference for the retraction notice.

**Response:** We are thankful for your suggestion.

- We found that the reference no. 30 is a duplicated of no.5, therefore; we deleted the reference no. 30 and corrected the reference no. within the main text. (Line 249)
- The reference no. 20 is only an abstract. We changed the reference style followed the Vancouver referencing style for abstract. (Line 343-347)
- Few corrections of format of the reference no. 10 and 14.

**From Reviewer 1:**

1. Line 153-155, it is not clear to me how the sample size of 21 is determined with using various \rho values. Please specify.

**Response:** As we clarified that we do not have an information about $\rho$ but we believed that the correlation should be more than 0.5 because we planned to measure the radiation dose in the same individual. We calculated the sample size with $\rho$ from 0.5-0.8 (showed in the table) and selected to use the highest sample size number from lung using $\rho$ 0.5.

We also added more information about sample size calculation into statistical analysis subsection (Line 153-156)

| **Heart**  **SD=1.4** | $\rho$ | **N** |
| --- | --- | --- |
|  | 0.5 | 11 |
|  | 0.6 | 9 |
|  | 0.7 | 6 |
|  | 0.8 | 4 |
| **Lung**  **SD=2.02** | $\rho$ | **N** |
|  | 0.5 | **21** |
|  | 0.6 | 16 |
|  | 0.7 | 12 |
|  | 0.8 | 8 |

2. Line 183-184, ' Moreover, using ACSB resulted in a significant decrease in mean heart dose (MHD) with 9.17 vs 1839.81 Gy (p<0.0001) and maximum heart dose with 43.79 vs 45.45 Gy (p=0.0144).' I think here more accurately, the 'mean heart dose' should be the mean of the mean heart dose and the 'maximum heart dose' is 'the mean of the maximum heart dose'.

**Response:** Thank you for your comment. We modified the manuscript followed your recommendation.

- Line 184-185
- Line 244-245
- Line 254

3. Line 187, 'no difference in the LAD volume' should be 'no statistical difference in the mean LAD volume'. There are some other places with similar problems that I would suggest the authors consider for a more accurate expression.

**Response:** Thank you and we agree with your suggestion. We already made a change of our manuscript as your suggestion.

- Line 189
- Line 199
- Line 234

4. Figure 2, please add units to x and y axis labels.

**Response:** We are grateful to the reviewer for noticing this error and already add units of the x and y axis in figure 2.

Sincerely,

Pitchayaponne Klunklin, MD
